# Progressive Osteolysis After Use of Synthetic Bone Graft Substitute

**DOI:** 10.7759/cureus.20002

**Published:** 2021-11-29

**Authors:** Punnavit Harimtepathip, Lamar F Callaway, Margaret A Sinkler, Suash Sharma, Kelly C Homlar

**Affiliations:** 1 Orthopedic Surgery, Augusta University Medical College of Georgia, Augusta, USA; 2 Medicine, Augusta University Medical College of Georgia, Augusta, USA; 3 Hematology and Oncology, Augusta University Medical College of Georgia, Augusta, USA

**Keywords:** bone graft substitutes, synthetic bone graft, osteolysis, benign bone tumor, pro-dense, fibrous dysplasia, polymethyl methacrylate, calcium sulfate, bone cement, calcium phosphate

## Abstract

Benign bone tumors are commonly treated with intralesional curettage and bone graft, with autogenous bone graft being the gold standard. However, autogenous bone graft has its limitation, and artificial bone graft substitutes were developed as an alternative. PRO-DENSE™ (Wright Medical Technology, Arlington, Tennessee) is a calcium sulfate and calcium phosphate mixed bone graft substitute that is biodegradable and osteoconductive, which has made them a popular choice among surgeons. However, long-term studies of this treatment method for benign tumors are still limited.

In this report, we present a case of progressive femoral neck osteolysis caused by an inflammatory reaction to PRO-DENSE™ two years after intralesional curettage and bone grafting of a benign bone tumor.

A twenty-one-year-old female with fibrous dysplasia underwent intralesional curettage with the use of PRO-DENSE™ bone substitute to fill the cavitary defect. She developed an inflammatory reaction to the bone graft substitute leading to increasing pain and osteolysis requiring a reoperation.

Bone graft substitute has many advantages; however, they should be used with discretion due to many unknown regarding their safety and long-term outcomes.

## Introduction

Benign bone tumors are commonly treated with intralesional curettage. Although controversy exists with regards to the augmentation of the remaining osseous defect, bone grafting remains a reasonable option [1-3]. The gold standard for all grafting procedures is autogenous bone graft; however, due to limited supply and donor site morbidity, artificial bone graft substitutes were developed as an alternative [4]. One synthetic bone graft substitute is PRO-DENSE™ (Wright Medical Technology, Arlington, Tennessee), which is composed of calcium sulfate and calcium phosphate matrix mixed with beta-tricalcium phosphate granules [5]. PRO-DENSE™ and similar bone graft substitutes have advantages of being abundant in supply, biodegradable, and osteoconductive, which has made them a popular choice among surgeons. However, long-term studies of this treatment method for benign tumors are still limited. Despite advances in bone graft substitute composition, complications directly related to implanted synthetic bone graft continue to occur, many of which are related to local inflammatory reactions [6-8].

In this report, we present a case of progressive femoral neck osteolysis caused by an inflammatory reaction to PRO-DENSE™, two years after intralesional curettage and bone grafting of a benign bone tumor. The patients and/or their families were informed that data from the case would be submitted for publication and gave their consent.

## Case presentation

**Figure 1:**
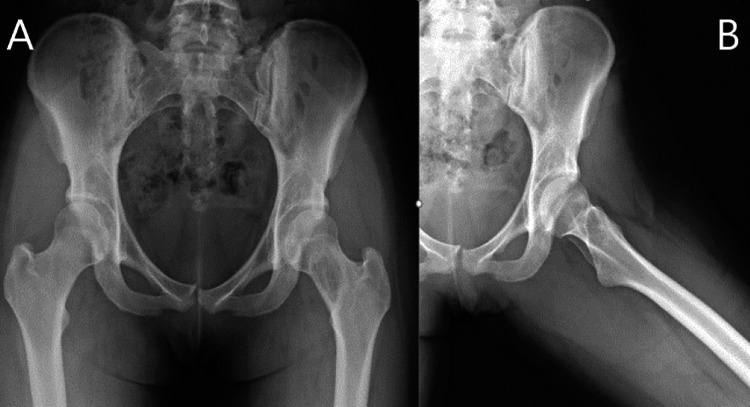
Radiographic anteroposterior (A) and frog-leg lateral (B) views of the hip from initial presentation revealed lesion within the left femoral neck

**Figure 2:**
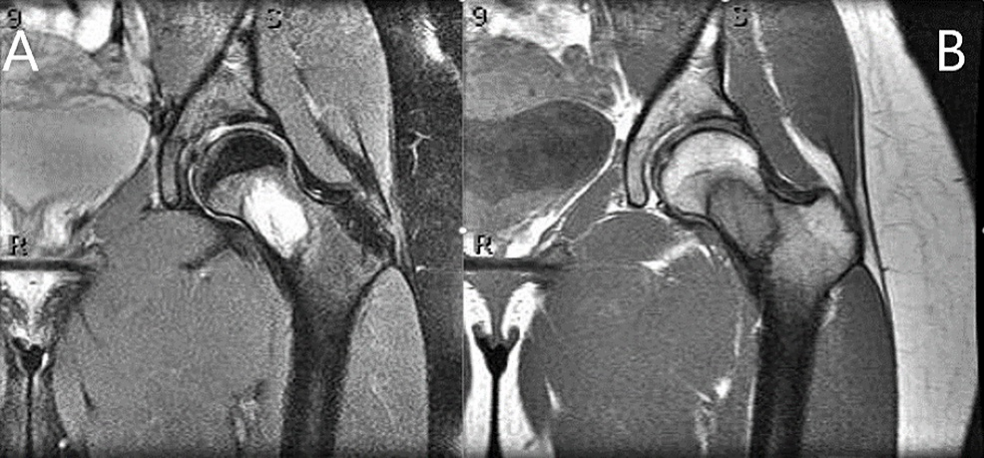
MRI scan of the left hip MRI scan of the left hip with T2-weighted coronal image (A) demonstrating a hyperintense isolated lesion with distinct sclerotic margins and surrounding bone marrow edema within the femoral neck and a T1-weighted coronal image (B) showing an isointense lesion with sclerotic margins

**Figure 3:**
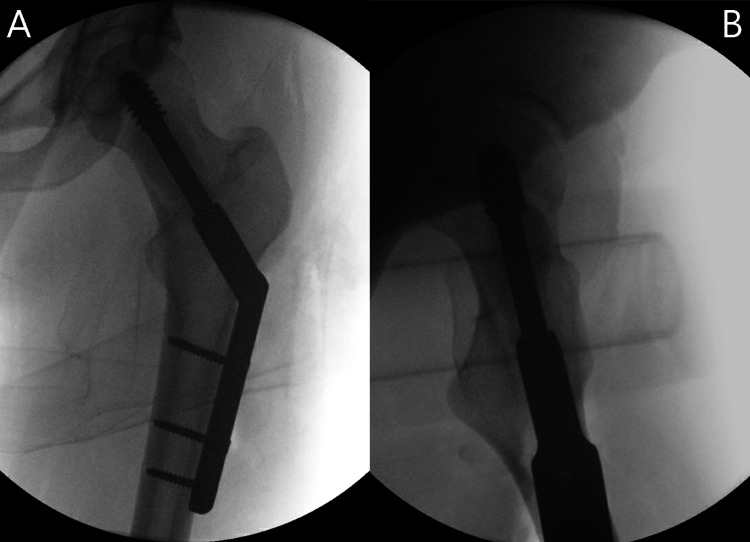
Radiographic anteroposterior (A) and lateral (B) views of the left hip intraoperatively after curettage with use of PRO-DENSE™ and operative fixation

A twenty-one-year-old female with no significant past medical history presented to the clinic with a three-year history of left groin pain made worse with weight-bearing. Initial radiographs showed an approximately four-centimeter lesion within inferomedial femoral neck, surrounded by a reactive sclerotic border. MRI showed an isolated lesion with distinct margins within the femoral neck, which was isointense T1, hyperintense on T2 with some internal mineralized areas and reactive sclerotic border, a large amount of surrounding edema in proximal diaphysis up to the physis, suggestive of maturing fibro-osseous lesion/fibrous dysplasia (Figures 1 and 2). Despite extensive conservative treatment, she continued to have pain with weight-bearing to the point it was limiting normal daily activities. An image-guided core needle biopsy was performed and was consistent with fibrous dysplasia. Given her persistent pain, she was taken for open biopsy, intralesional curettage, and prophylactic stabilization of the left femoral neck with a dynamic hip screw. Twenty cubic centimeters of PRO-DENSE™ were placed within the osseous defect (Figure 3).

**Figure 4:**
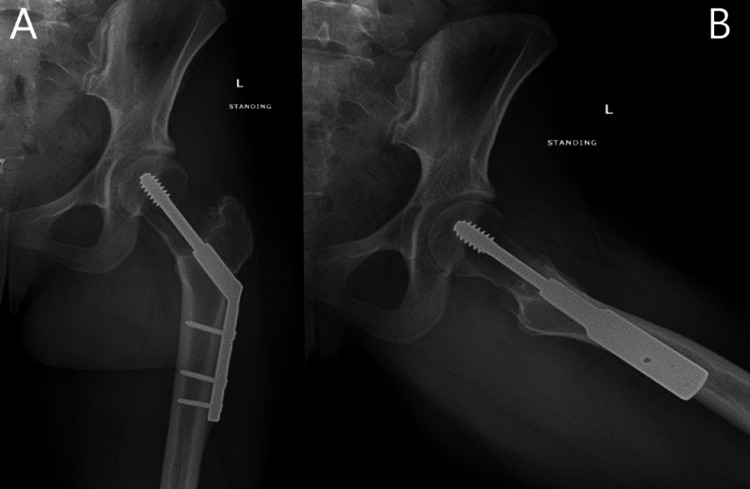
Radiographic anteroposterior (A) and frog-leg lateral (B) views of the left hip reveal progressive osteolysis within the femoral neck in the area of previous bone graft placement

**Figure 5:**
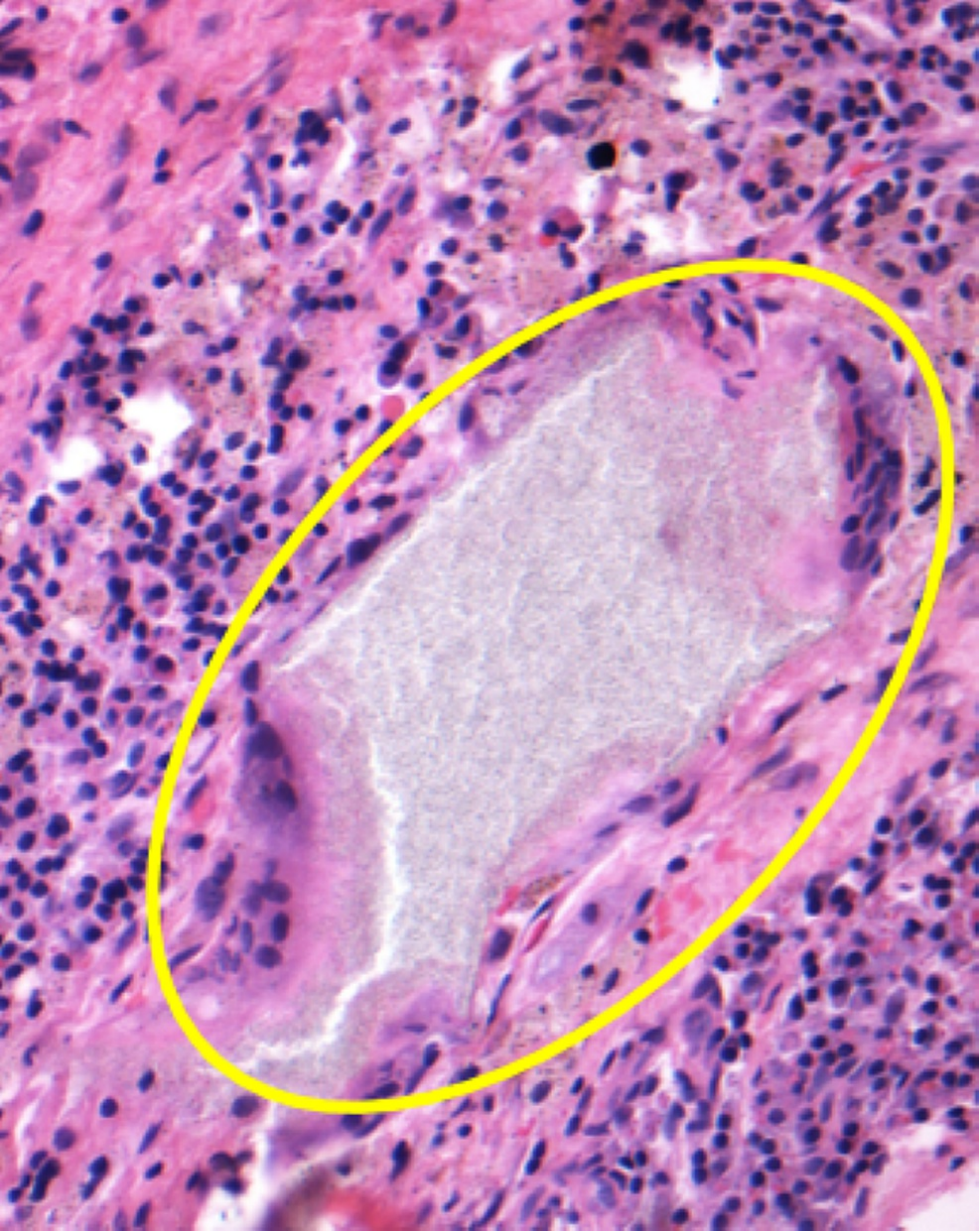
Hematoxylin and eosin staining at 200 magnification showing a large deposit of foreign material with associated foreign-body type giant cell reaction and surrounding chronic inflammation

**Figure 6:**
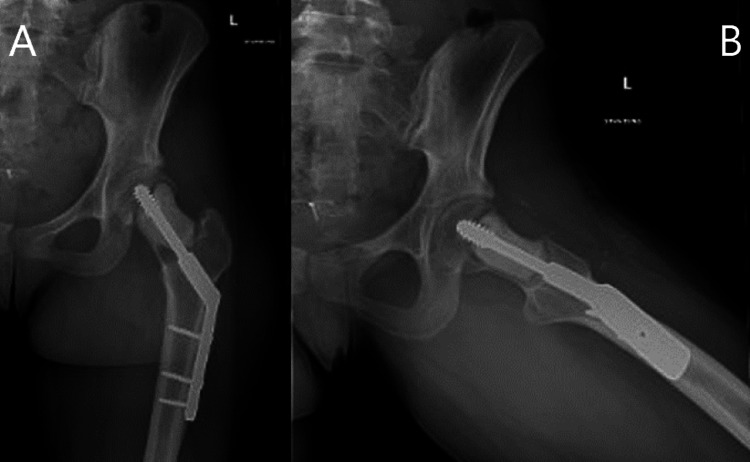
Postoperative radiographic anteroposterior (A) and frog-leg lateral (B) views of the left hip after curettage and PMMA placement PMMA - polymethylmethacrylate

**Figure 7:**
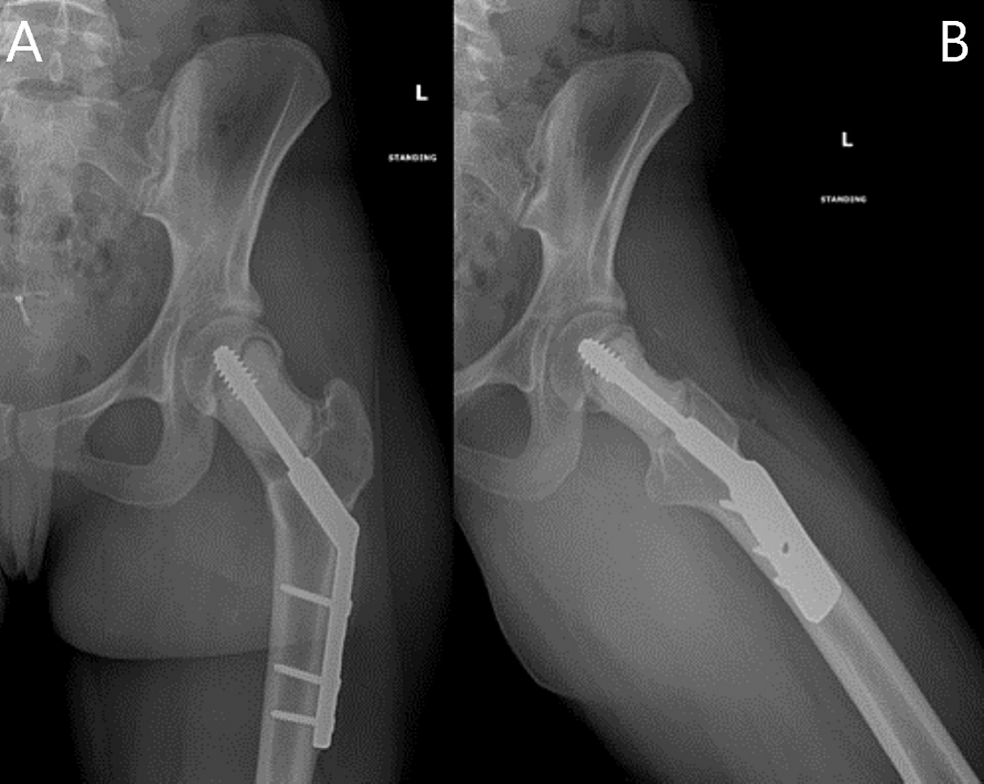
Radiographic anteroposterior (A) and frog-leg lateral (B) views of the left hip taken at one-year follow up reveal no areas of osteolysis

The patient did well initially but began having increasing pain. Serial radiographs showed expected centripetal resorption of the graft, all of which was resorbed by five months; however, the patient continued to have progressive osteolysis two years postoperatively (Figure 4). Given the extent of osteolysis and disruption of the anterior cortex, she was referred for an image-guided core needle biopsy to confirm the original diagnosis and that no secondary lesion was present. Biopsy showed a hypocellular specimen with few macrophages, which was negative for malignancy with negative cultures. An inflammatory reaction to the bone graft substitute was suspected, and open biopsy via an anterior approach to the hip, intralesional curettage, and placement of polymethylmethacrylate (PMMA) bone cement was performed. Intraoperative biopsy showed fragments of fibro-connective stroma and remodeled bone with chronic inflammation, hemosiderin, scar fibrosis, and foreign-body giant cell reaction (Figure 5). Special stains for bacteria, acid-fast bacillus (AFB), and fungal organisms were negative, with no malignancy or residual fibrous dysplasia identified. Post-operative images from the curettage and PMMA placement were obtained (Figure 6). Following the second procedure, the patient progressed as expected. She had significant improvement in pain and was able to return to all previous activities. Radiographs at one-year follow-up from her second operation revealed stable implants with no evidence of progressive osteolysis (Figure 7).

## Discussion

Following intralesional curettage of benign bone tumors, bone graft is often used to fill the osseous defect in an attempt to accelerate bony ingrowth and allow for possible earlier weight-bearing [9]. While autograft is historically the gold standard for bone substitution, bone graft extenders and substitutes are abundant in supply, readily available, and avoid complications from the use of autograft or allograft, such as donor site pain and immunogenic reactions, respectively [10]. Although often convenient, bone graft substitutes are not without unique complications. The most common complications reported that are directly attributable to the use of synthetic bone grafts are pain, delayed wound healing, sterile inflammatory reaction, prolonged wound drainage, and soft tissue cystic formation [6-8]. All of these complications are thought to be caused by inflammatory reactions to the bone graft, possibly related to the rapid resorption of calcium sulfate [11].

Friesenbichler et al. reported an adverse reaction to geneX, a bone graft substitute comprised of a β-tricalcium phosphate/calcium sulfate hemihydrate compound, used in bone defect reconstruction after intralesional curettage of benign and low-grade malignant bone tumors resulting in wound complications such as marked local aseptic inflammation of muscle and skin, and inflammatory cystic formations. No identifiable associated factors such as smoking or known allergies could be linked to these reactions [11]. Robinson et al. noted an inflammatory reaction, following calcium sulfate use, on MRI three days postoperatively which resolved without complication or intervention in two months. This inflammation may be due to rapid graft resorption resulting calcium-rich fluid and lowered pH [6].

Civinini et al. reviewed the kinetics of calcium sulfate/calcium phosphate bioceramics with use of quantitative CT which revealed that these bioceramics can persist for up to two years which is consistent with the findings in our case [12]. Unlike prior reports, we provide histologic evidence of an adverse reaction to bone graft substitute causing progressive osteolysis. Histologic evaluation of the intra-operative specimen revealed histiocyte rich chronic inflammation and a few giant cells with both polarizable and nonpolarizable foreign material which we believe was a reaction to the ceramic bone graft substitute, PRO-DENSE™, used in the initial procedure. In our case, this inflammatory reaction to the bone graft substitute led to an increased area of osteolysis around the implant in the femoral neck region, as seen in the postoperative radiograph. This leads to weakening of the osseous integrity and increases the risk for serious complications such as implant loosening, femoral neck fracture, avascular necrosis, and collapse of the articular surface which may necessitate the need for arthroplasty in a young patient.

An additional shortcoming of synthetic bone graft substitutes is the graft incorporation profile. Many of these products contain calcium sulfate which is absorbed before bony ingrowth occurs. The radiolucency observed on postoperative radiographs can be difficult to interpret as recurrence or normal resorption. There is typically a predictable pattern of absorption and osseous ingrowth, which progresses from the periphery towards the center [13,14]. Auston et al. discusses unexpected radiographic lucency (URL) following the use of calcium sulfate/tricalcium phosphate bone graft substitutes [9]. They describe URL as any radiolucency that varied from the typical pattern of osseous ingrowth and occurred in 13% of patients in their study. The average time to identification of the URL was 27 weeks. They were unable to identify the specific etiology but proposed URLs could be related to inflammatory or foreign body reactions and ultimately concluded that URLs do not result in clinically relevant adverse events [9]. In our case, we initially noticed a URL at three months postoperatively, but unlike URLs previously described, this radiolucency continued to enlarge two years postoperatively (Figure 4), causing significant pain and increasing the risk of serious complication, ultimately led to an additional operation two years after the initial procedure.

## Conclusions

In conclusion, bone graft substitutes have many advantages, including decreased donor site morbidity, cost, and availability. However, there are many unknowns about their safety, efficacy, and long-term outcomes. Bone graft substitutes are viable alternatives to autograft and allografts; however, they should be used with discretion. Our case report represents a clinically significant osteolytic reaction to bone graft substitute requiring an additional operative intervention and places the patient at increased risk for serious complications. Future studies will provide a more appropriate understanding of the risks and benefits of its utilization.
